# Three-dimensional morphological analysis of the femoral neck torsion angle—an anatomical study

**DOI:** 10.1186/s13018-020-01712-8

**Published:** 2020-05-27

**Authors:** Ru-Yi Zhang, Xiu-Yun Su, Jing-Xin Zhao, Jian-Tao Li, Li-Cheng Zhang, Pei-Fu Tang

**Affiliations:** 1 Medical School of Chinese PLA, No. 28, Fuxing Road, Beijing, 100853 China; 2 Department of Orthopedics, Shijingshan Teaching Hospital of Capital Medical University, Beijing Shijingshan Hospital, No. 24, Shijingshan Road, Beijing, 100043 China; 3 Department of Orthopedics, Chinese PLA General Hospital, National Clinical Research Center for Orthopedics, Sports Medicine & Rehabilitation, Beijing, 100853 China

**Keywords:** Femoral neck torsion angle (FNTA), Femoral neck isthmus (FNI), Femoral neck basilar part (FNB), Coronal plane of the proximal femur, Morphology

## Abstract

**Background:**

The femoral neck torsion angle (FNTA) is an important but often neglected parameter in assessments of the anatomical morphology of the femoral neck, which is often confused with the femoral neck anteversion angle (FNAA) in the current literature. Currently, the measurement methods reported in the literature all adopt the naked eye or two-dimensional (2D) visualization method, and the measurement parameters and details are not clearly defined. The objection of this research was to provide a reliable 3D method for determining the femoral neck axis, to improve the measurement method of the FNTA, and to analyze the anatomical and clinical significance of the results.

**Methods:**

Computed tomography (CT) data of 200 patients who received a lower extremity CT angiography examination were selected, and the bilateral femurs were reconstructed with three dimensional CT (3D CT). First, the 3D axis of the femoral neck was built. Second, the long axis of the cross section the femoral neck isthmus (FNI) and femoral neck basilar part (FNB) were confirmed by the “inertia axes” method, and the plane consisting of the long axis of the cross-section and the center of the femoral head was defined as the long axial plane. Third, the coronal plane of the proximal femur was determined through the long axis of the proximal femur and the femoral coronal. Finally, the FNTAs (the angles between the long axial planes and the coronal plane of the proximal femur) of FNI and FNB were measured. The size of FNTA was compared between the sexes and sides and different locations, the correlation between the parameters and age, height, and weight were evaluated.

**Results:**

The difference in FNTA was statistically significant between the isthmus and the basilar part (isthmus 30.58 ± 8.90° vs. basilar part 23.79 ± 3.98°; *p* < 0.01). Significant difference in the FNTA was observed between the sexes (males 31.99 ± 9.25° vs. females 27.49 ± 7.19°; *p* < 0.01). The increase in FNTA from the basilar part to the isthmus was 6.79 ± 8.06°, and the male (7.87 ± 8.57°) was greater than the female (4.44 ± 6.23°, *p* < 0.01). However, no significant difference in the values was observed between sides. Height exerted the greatest effect on the FNTA according to the correlation analysis (*r* = 0.255, *p*< 0.001).

**Conclusions:**

This study found a reliable 3D method for the determination of the femoral neck axis improved the measurement method of the FTNTA and made it more accurate and repeatable. The results provided a methodological basis and theoretical support for the research and development of internal fixation device for femoral neck fracture and the spatial configuration of implants in treatment. And the optimal opening point of the femoral medullary cavity was recommended to locate at the posterior position of the top of the femoral neck cross-section during hip replacement.

## Introduction

The anatomical morphology of the femoral neck plays an important role in the recognition and treatment of diseases around the hip joint. Many morphological parameters (the femoral neck-shaft angle, femoral neck anteversion angle (FNAA), and so on) are closely related to the findings in clinical studies [1–6]. However, Kate suggested that femoral neck torsion angle (FNTA) and FNAA were two different angles in1976. But prior to this, FNTA was an underestimated anatomical parameter [7]. The current studies have found that the FNTA has important clinical significance in determining screw space configuration for internal fixation of femoral neck fractures, the screw hole design of the proximal femoral neck plate, and the proximal femoral medullary opening point and femoral prosthesis placement during hip joint replacement [6, 8–12]. Therefore, the accurate definition and measurement standard of the FNTA are important.

According to the study by Kate, the FNTA was defined as the angle which formed by the femoral neck rotating around its axis and was different to the FNAA (the angle formed by the femoral neck rotating around the proximal femur axis), but the measurement of FNTA was performed using a two-dimensional (2D) method. Zhu et al. [13] suggested the use of a computed tomography (CT) to reconstruct 30 pairs of femur to distinguish the FNTA from the FNAA in his study. However, in their study, the position and direction of the femoral neck cross-section and the proximal femoral coronal plane were not clearly defined, which will directly affect the measurement results of the FNTA. In the present study, an accurate and reliable 3D measurement method for the FNTA was established, through defining the position and direction of the cross-section of femoral neck and the proximal coronal plane of femur precisely (details are provided Fig. 2 and Fig. 3). The size of the FNTA at different cross-sections (femoral neck isthmus (FNI) and femoral neck basilar part (FNB)) in 200 patients was measured using this method, the size of FNTA was compared between the sexes and sides, and the correlation between the parameters and age, height, and weight were evaluated, thus providing a reference for further clinical applications and research.

## Materials and methods

CT data of 213 patients who received a lower extremity CT angiography examination in our hospital from December 2009 to December 2012 were collected. Two hundred cases met the inclusion criteria, including 137 men and 63 women. The age ranged from 50 to 85, with an average age of 69.41 ± 9.21 years. Inclusion criteria were patients (1) older than 18 years (2) who did not present with femoral head necrosis, (3) severe hip osteoarthritis or rheumatoid arthritis, (4) a hip joint or femur deformity, (5) a history of hip or femur fractures, or (6) a history of hip or femur surgery. This research project was approved by the ethics committee of Chinese PLA General Hospital. As the study was a retrospective survey of medical imaging data and the anonymity of the patients’ data was maintained, informed consent was not required from patients.

All CT data were collected from the same CT machine (Siemens AG, Erlangen, Germany) with the same scanning parameters (120 KV; 210 mA; collimation, 4 mm; table speed, 3–5 mm/s; and number of slices, 80–100). The slice thickness of CT scans analyzed in this study was 1.2 mm. The 3D models of femur were reconstructed by the threshold segmentation and the interactive editing method in the Mimics software (version 12.0, Materialise, Leuven, Belgium), and a standardized coordinate system for each femoral model was constructed using the method described by Su et al. [14], and the coronal, sagittal, and horizontal planes were defined to avoid interference from body position during the measurement of FNTA. The reconstructed femur model was input into the 3-Matic software (Materialise N.V., Belgium) in STL format, which geometry is triangle mesh.

**Fig 1.**
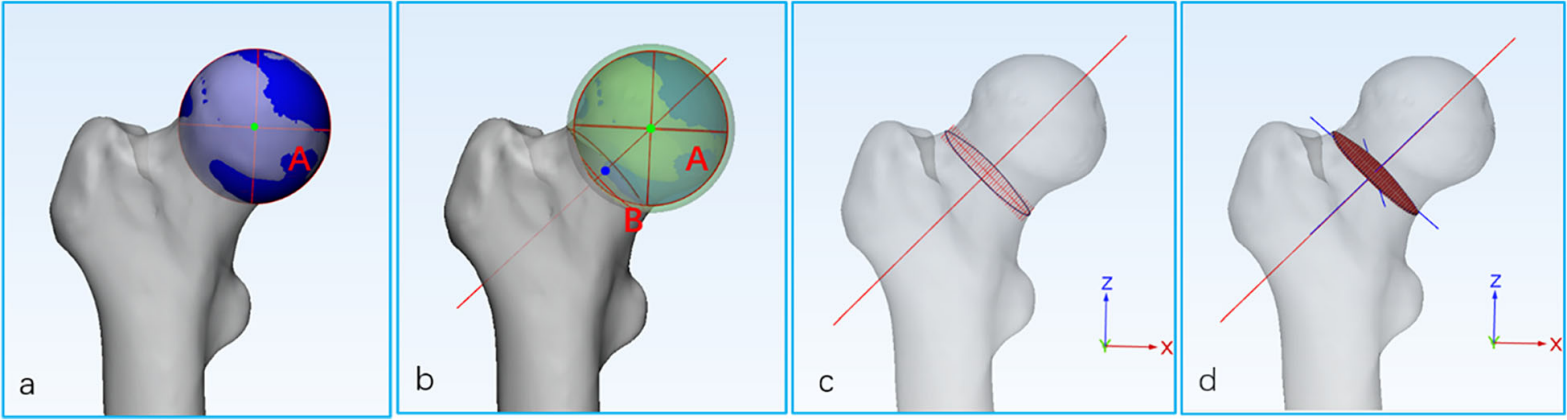
**a–d** The method for determining the 3D axis of the femoral neck and the “inertia axis” method. **a** The femur head was simulated as a closed sphere (blue) and the center of the sphere was defined as the center of the femur head, namely, point A. **b** A concentric (point A) sphere (green) was generated by increasing the radius of the sphere fitted to the femoral head by 2 mm, which cut the femoral neck to obtain a corresponding cross-section. This cross-section was treated as a fitting circle, with the center defined as point B. Finally, the connecting line between point A and B was considered the 3D axis of the femoral neck. **c** The 3D axis of the femoral neck and cross-section of the FNI. **d** The cross-section of the FNI was extruded to a 0.5-mm depth, and then the inertia axes (three blue lines) of the extruded part of the cross-section of the FNI were established using the “fit inertia axes” method in 3-Matic software

First, the femoral head surface was marked using the “Wave Brush Mark” method in the software, then the marked triangles of femur head was created a sphere using the “Analyze” method in the software [15]. The center of the sphere was defined as the center of the femur head, namely, point A. Second, point A as the center of the original sphere, its radius was increased by 2 mm to generate a solid ball which can fully contain the entire femoral head and just tangent to the femoral neck isthmus observed with the naked eye, according to the preliminary experiment. The generated solid ball cut the femoral neck to obtain a corresponding section. This section was treated as a fitting circle, with the center defined as point B. Finally, the line connecting point A and point B was defined as the 3D axis of the femoral neck (Fig. 1a, b).

A series of continuous vertical sections was established along the axis of the femoral neck with an interval of 1 mm between adjacent sections. The software automatically generated the area of each section, and the smallest cross-section of three adjacent minimum cross-sections was defined as the FNI. The position of the anterior cross-section in which the femoral neck is connected to the greater or lesser trochanter was defined as the FNB. The cross-sectional morphology of the femoral neck was reported as oval-like shape by morphological study [16, 17]. In this study, the cross section of the femoral neck was generated into a part with a thickness of 0.5 mm. Two lines located on the cross-section of the three inertial axes of the part were defined as the long axes (from anterior top to the posterior bottom of the femoral neck) and short axes (from the posterior upper part to the anterior lower part the femoral neck) of the cross section of FNI and FNB. The method used to determine the long and short axes was defined as the “inertia axis” method (Fig. 1c, d).

**Fig. 2:**
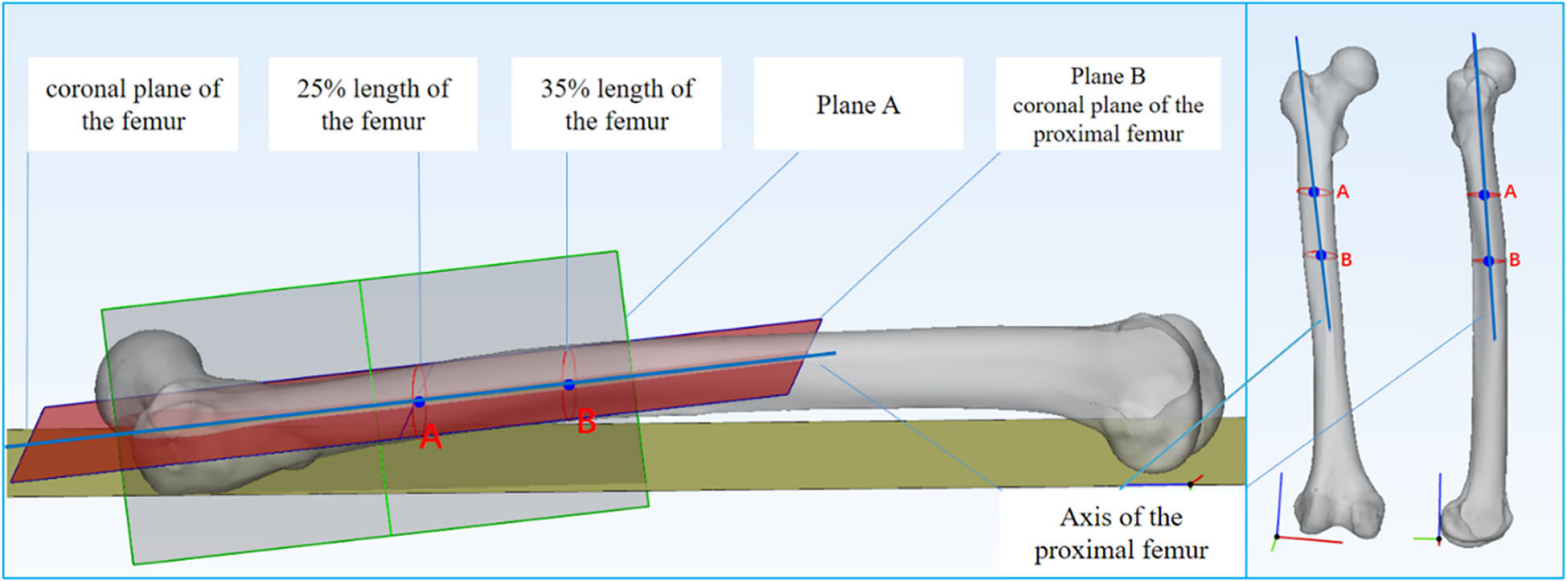
The method for determining the coronal plane and axis of the proximal femur. The blue line through points A and B (the centers of the inner connecting circles of these two cross-sections represent 25% and 35% of the length of the femur shaft) was defined as the axis of the proximal femur. The gray plane perpendicular to the coronal plane (yellow) of the femur through points A and B was defined as plane A, and then a plane perpendicular to plane A was defined as plane B (red), which was also designated the coronal plane of the proximal femur

**Fig. 3:**
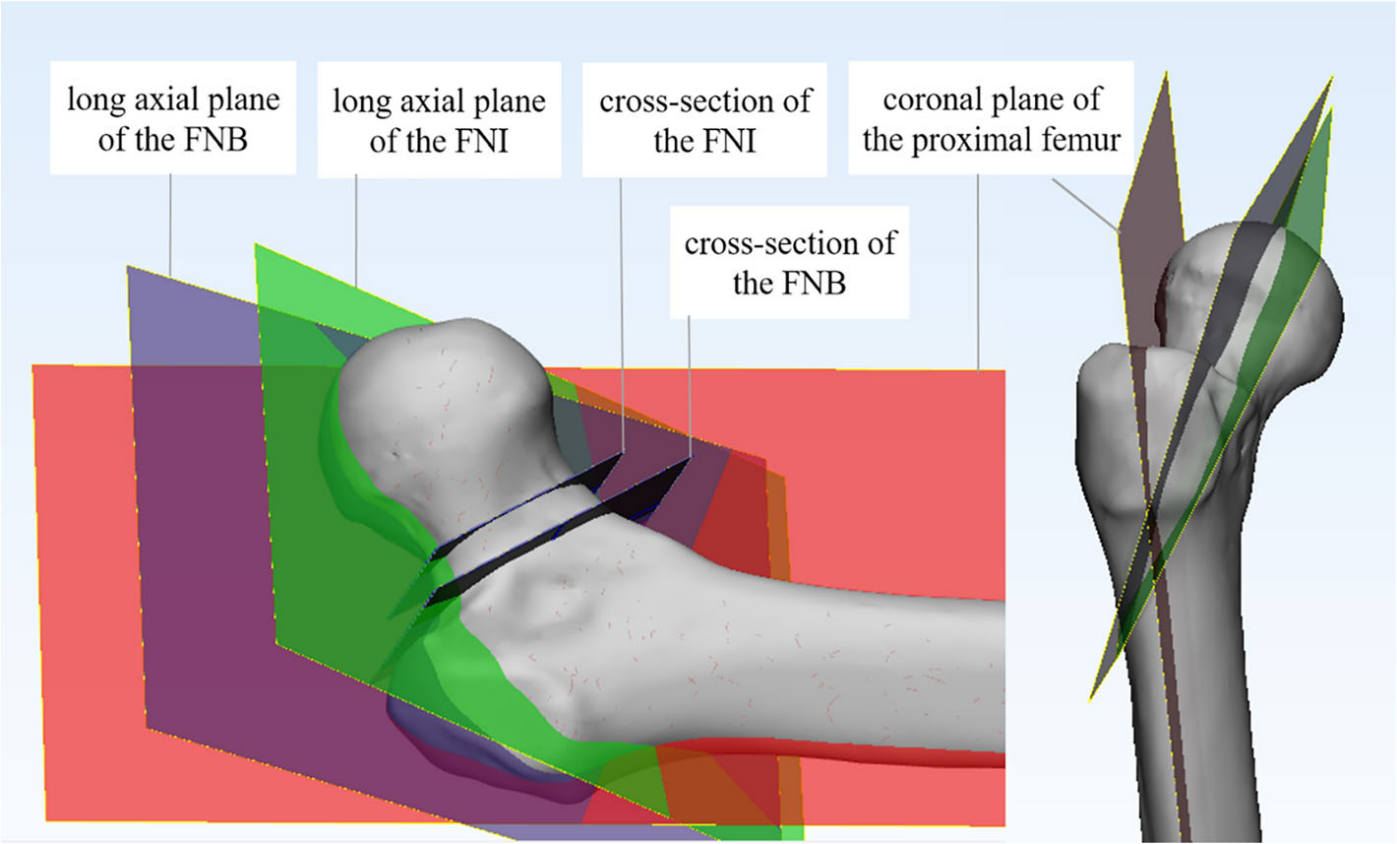
The long axial plane. The red plane is the coronal plane of proximal femur, and the positions of the FNI and FNB are intersected by two black planes. The green plane is the long axial planes of the FNI, and the blue plane is the long axial plane of the FNB.

**Fig. 4:**
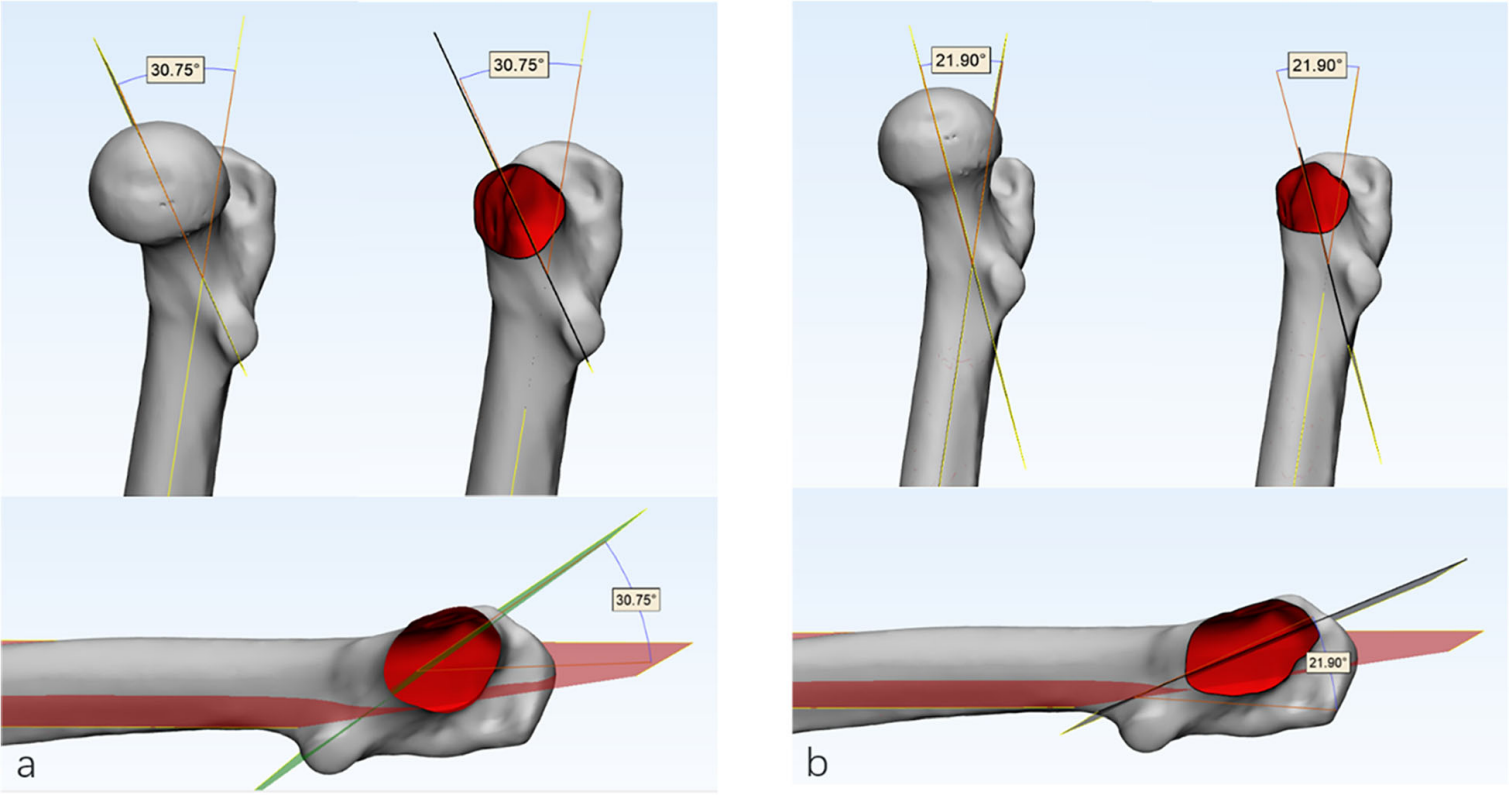
**a** The FNTA of the isthmus (30.75°)**. b** The FNTA of the basilar part (21.90°)

At the proximal femur, 25% and 35% of the femoral shaft length, cross-sections of the femur were created after the intersection of the femur with the transverse plane [1]. Then, the inner connecting circles of these two cross-sections were created, and the centers of these two circles were obtained. The line through the centers was defined as the axis of the proximal femur, which was distinct from the axis of the femur. The latter was not a straight line but a curve due to the anterior and lateral arch of the femur [18]. Using 3-Matic software, a plane perpendicular to the coronal plane of the femur through these two centers was defined as plane A, and then a plane perpendicular to plane A was defined as plane B, which was also named as the coronal plane of the proximal femur (Fig. 2). According to the method introduced by Zhu et al. [13], the plane consisting of the long axis of the FNI cross-section and the center of the femoral head was defined as the long axial plane of the FNI, and the plane consisting of the long axis of the FNB cross-section and the femoral head center was defined as the long axial plane of the FNB (Fig. 3). The FNTAs of the isthmus and basilar part were defined as the angles between the long axial planes of FNI and FNB and the coronal plane of the proximal femur, which were measured directly using 3-Matic software (Fig. 4). The difference between the isthmus FNTA and the basilar FNTA was defined as the increase in the FNTA (iFNTA).

The intraclass correlation coefficient (ICC) was used to assess the reliability of the measurement method established in the present study. The sample size required in the reliability study was calculated using the formula reported by Walter and Eliasziw [19]. Subsequently, three observers and another observer made three repeated measurements of any 15 pairs of femur samples. Based on the suggestion proposed by Weir [20], a repeated-measures ANOVA was applied to avoid a significant difference in the results of the study. Two-way random and two-way fixed models were used to evaluate inter- and intraobserver reliability [21]. Fifteen paired samples were subjected to repeated FNTA measurements in a random order by one senior attending orthopedic doctor (RYZ) with a minimum of a 24-h interval between trials to evaluate the intraobserver reliability. The same measurements on the same specimens were performed in an independent manner and a random order to assess interobserver reliability by three other doctors (XYS, JXZ, and JTL).

The measured data were analyzed using IBM SPSS Statistics software for Windows, Version 21.0 (IBM Corp., Armonk, NY, USA). Pearson’s correlation coefficients (normal distribution) or Spearman’s rank correlation coefficients (Non-normal distribution) were calculated to analyze potential relationships between demographic data (age, height, weight, and BMI) and the FNTA, according to whether the measured data is normally distributed. A paired *t* test was used to compare the FNTA between the isthmus and the basilar part, and the FNTA in all subjects was analyzed using a two-way ANOVA. A stepwise linear regression model was applied to investigate the factors influencing the FNTA. Statistical significance was established at *p* < 0.05.

## Results

The main characteristics (demographic data) of the participants and the differences between the sexes were summarized. The difference in age between male (69.27 ± 9.50 years) and female (69.68 ± 8.55 years) patients was not statistically significant (*p* = 0.513), but statistically significant differences in height (males 1.68 ± 0.06 m vs. females 1.59 ± 0.06 m; *p* < 0.01), weight (males 66.24 ± 8.81 kg vs. females 62.32 ± 9.80 kg; *p* < 0.01) and BMI (males 23.39 ± 2.70 kg/m^2^ vs. females 24.77 ± 3.54 kg/m^2^; *p* < 0.01) were observed.

High intraobserver and interobserver reliability (*n* = 30) were observed, with ICC values of 0.989 and 0.996, respectively, and the mean squares within trials ranged from 0.131 to 0.179, with all *p* values were greater than 0.05 (Table 1). The FNTA of the isthmus was larger than the basilar part in different groups, and the difference was statistically significant (Table 2). The FNTAs were significantly different between the sexes, with significantly greater values recorded in men than in women (*p* < 0.05). No statistically differences were observed between sides or between the sexes and side interactions (Table 3).

**Table 1:** Intraobserver and interobserver reliability of the measurements

Items	Intraobserver reliability		Interobserver reliability	
	ICC	95% CI	ICC	95% CI
Isthmus FNTA	0.993	0.989–0.996	0.995	0.991–0.998
Basilar FNTA	0.989	0.979–0.994	0.996	0.990–0.998
iFNTA^#^	0.991	0.983–0.996	0.995	0.989–0.998

^#^ iFNTA The difference between the isthmus FNTA and the basilar FNTA

*ICC* The intraclass correlation coefficient, *CI* confidence interval

**Table 2:** Paired-sample *t* test of the FNTA (mean ± SD°)

	Total (400)	Males (137)	Females (63)	Left (200)	Right (200)
Isthmus FNTA	30.58 ± 8.90	31.99 ± 9.25	27.49 ± 7.19	30.06 ± 8.57	31.10 ± 9.21
Basilar FNTA	23.79 ± 3.98	24.13 ± 4.00	23.05 ± 3.84	23.49 ± 4.01	24.10 ± 3.92
T value	16.834	15.186	7.995	12.365	11.529
*P* value	< 0.001	< 0.001	< 0.001	< 0.001	< 0.001

**Table 3:** Differences in the FNTA (mean ± SD, °) between sexes and sides (P1 value for sexes; P2 value for sides; P3 value for the interaction between sex and side)

Items	Males (137)		Females (63)		P1	P2	P3
	Left	Right	Left	Right			
Isthmus FNTA	31.37 ± 8.92	32.62 ± 9.56	27.20 ± 6.98	27.78 ± 7.44	< 0.001	0.328	0.722
Basilar FNTA	23.92 ± 3.97	24.34 ± 4.03	22.55 ± 3.99	22.55 ± 3.64	0.011	0.095	0.495
iFNTA^#^	7.45 ± 7.94	8.27 ± 9.18	4.65 ± 6.12	4.23 ± 6.38	< 0.001	0.812	0.466

The results of the correlation analysis revealed positive correlations between the isthmus FNTA and iFNTA with height, and between the basilar FNTA and iFNTA with body weight; only the basilar FNTA was negatively correlated with BMI. All correlation coefficients were shown in Table 4. A stepwise linear regression analysis was conducted with age, height, weight, and BMI as independent variables to determine the most relevant factor that affected the FNTA. Ultimately, height exerted the greatest effect on the FNTA, and the final regression model of the isthmus FNTA was *Y* = − 27.685 + 35.134 × HEIGHT (*p* < 0.001, *R*^2^ = 0.095).

**Table 4:** The correlation (r value) between morphological parameters of the femoral neck and physical properties

	Age	Height	Weight	BMI
Isthmus FNTA	− 0.091	0.255^**^	0.061	− 0.102^*^
Basilar FNTA	− 0.018	0.050	− 0.169^**^	− 0.193^**^
iFNTA^#^	− 0.115^*^	0.262^**^	0.186^**^	0.098

*The correlation was significant at the level of 0.05 (two-tailed)

**The correlation was significant at the level of 0.01 (two-tailed)

## Discussion

The FNTA and FNAA are completely different anatomical measurements [7, 8, 13]. First, the former was defined as the angle between the long axial plane of the femoral neck cross-section and the coronal plane of the proximal femur, and the latter was defined as the angle between the 3D axis of the femoral neck and the coronal plane of the femur. Second, the sizes of the two angles differ from each other. Third, the results reported in the literature using the 3D CT measurement method showed that the FNAA is approximately 10°, while the FNTA is approximately 30° [7, 13]. Unfortunately, current studies often confuse the two angles [1, 6, 22]. In other words, the expression of the angles (femoral torsion angle and femoral neck torsion angle) was not standardized and consistent at present. For example, the expression of the FNTA was mentioned by Yin, Hartel, and Zhao, but in fact, it was actually the FNAA, according to the measurement method and results reported in their articles [1, 6, 22].

**Fig. 5:**
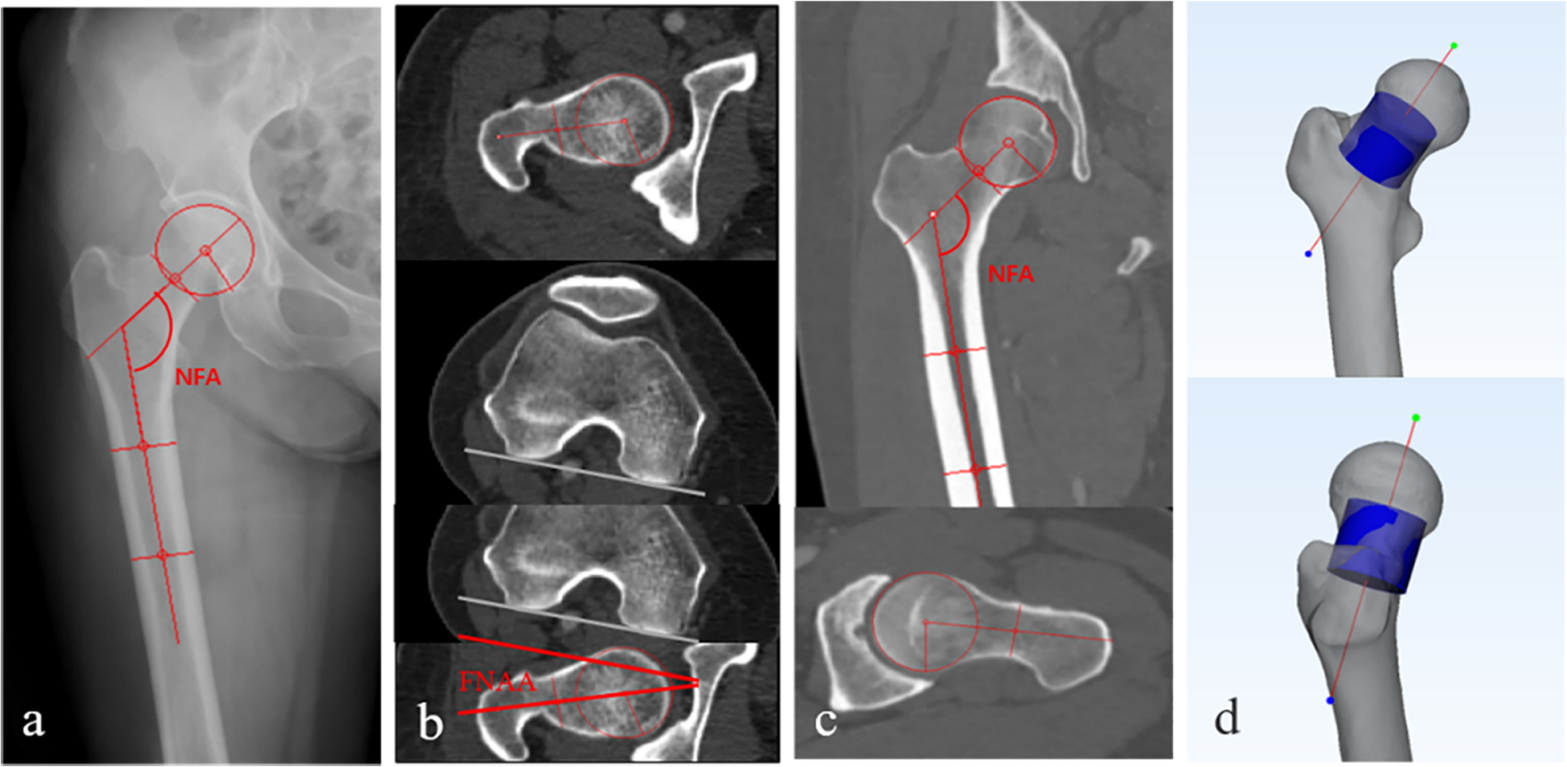
**a**–**d** The methods for defining the femoral neck axis. **a** The method described by Zhang YL et al. [3]. **b** The method described by Morvan et al. [4]. **c** The method reported by Nakanishi et al. [5]. **d** The method reported by Bonneau N et al. [16]

Many methods have been established to define the femoral neck axis. In the early stage, the axis of the femoral neck was determined by the anteroposterior and lateral centerline of X-ray or 2D CT, but both methods were affected by the femoral position during fluoroscopy, and the axis was ultimately two-dimensional axis [3, 4]. Nakanishi and Yin [5, 23] searched for the layers including both the femoral head and the femoral neck on coronal slices of 3D CT images, and they defined the connecting line between the femoral head center and the femoral neck isthmic center as the femoral neck axis. However, this method was also affected by the spatial position of the femur. Bonneau et al. [16] first proposed the concept of the 3D axis of the femoral neck. However, the reconstruction of the femoral neck medullary cavity is complicated because of the special distribution of bone trabeculae in the femoral neck (Fig. 4). In our study, the actual 3D axis of the femoral neck was generated using a 3D method. The shape of the femur is not a standard cylinder, the femoral trochanteric medullary cavity is irregular, and the femur length and curvature differ between men and women [16] (Fig. 5). Therefore, the present study adopted the method introduced by Hartel et al. [1] to determine the axis of the proximal femur. Based on the traditional coronal plane of the femur, the coronal plane of the proximal femur was created using the method of establishing a plane perpendicular to a specified plane through two points (details are provided in the “Methods” section).

The FNTA of the isthmus that we measured was very similar to that of the Kate (30°) and Zhu (31.34 ± 2.08°) reports, but these authors did not report the specific position of the femoral neck cross-section [7, 8]. Kate measured 1000 femur specimens in India, but the specific measurement method was not described in detail. Zhu et al. rebuilt the proximal femurs of 30 healthy adult volunteers and fitted the ellipse with the “concentric circle” method, but did not clearly define the position of the coronal plane of the proximal femur. Unfortunately, the lack of a definition in both of these articles significantly reduced the repeatability of their research methods. For the first time, the size of the FNTA at different positions (FNI and FNB) of the femoral neck was measured using 3-Matic software in the present study. The torsion of the femoral neck is not presumed to increase completely at one time from the FNB to FNI but may be increased gradually. The FNTAs at the FNI and FNB of the male patients are significantly greater than the female patients, which is of guiding significance for the treatment and posttreatment evaluation of patients of different sexes with femoral neck related diseases, such as the choice of the model of the internal fixation device. However, the FNTAs at FNI and FNB between left and right side were not significantly different, indicating that the anatomical morphology of the healthy side can be used as a reference for the treatment of the affected side in patients with femoral neck related diseases. Height exerted the greatest effect on the isthmic FNTA and the iFNTA in the present study, which may be related to local muscle strength, as more muscle strength may be needed to coordinate the posture of a taller individual [17].

Three cannulated screws in parallel are currently still the first choice for femoral neck fracture fixation [12, 24]. The presence of a torsion angle directly affects the nailing point and screw configuration on the lateral wall of the greater trochanter. Therefore, the spatial distribution of the three screws should match the morphology of the transverse plane (including the FNTA) of the femoral neck isthmus as much as possible to abut the screws to the femoral neck cortex without iatrogenic penetration and to obtain the maximum occupancy effect of the three screws [8, 9, 12, 25]. Similarly, the screw hole design of the proximal femoral plate should refer to the FNTA. The attachment of the plate should be satisfactory while reducing the penetration rate of the femoral neck screw [26, 27]. Due to the presence of the FNTA in basilar part, the long axis of the FNB cross-section was not located in the coronal plane of the proximal femur. Thus, forward deviation of the opening was likely to occur in the operation, resulting in difficult prosthesis placement, proximal femoral splitting, and periprosthetic fracture. Postoperative complications such as anterior femoral pain and early loosening of the prosthesis are common. Therefore, the optimal opening point of the femoral medullary cavity during hip replacement should be the posterior position of the top of the femoral neck cross-section [9–11].

This study has one limitation: the patients in this study were relatively old. Thus, the reference range of the measured morphological parameters does not represent the overall population. Studies examining an expanded age group or comparing the data with findings obtained from other research centers are necessary to circumvent this limitation.

## Conclusions

This study found a reliable 3D method for the determination of the femoral neck axis improved the measurement method of the FNTA and made it more accurate and repeatable. The FNTA of the isthmus was significantly greater than the FNTA of the basilar part. The size of the torsion angle of the neck isthmus of the femur was positively correlated with height and weight. The results of the FNTA measurement provided a methodological foundation and theoretical support for the research and development of internal fixation devices and the spatial configuration of implants in treatment of femoral neck fracture. And the optimal opening point of the femoral medullary cavity was recommended to be located at the posterior position of the top of the femoral neck cross-section during hip replacement.

## Data Availability

Data and materials were accessed from the case system of our department.

## Abbreviations

FNTA: Femoral neck torsion angle
FNAA: Femoral neck anteversion angle
2D: Two-dimensional
3D: Three-dimensional
CT: Computed tomography
FNI: Femoral neck isthmus
FNB: Femoral neck basilar part
iFNTA: Increase in the FNTA
ICC: The intraclass correlation coefficient
